# Cancer characterization using light backscattering spectroscopy and quantitative ultrasound: an ex vivo study on sarcoma subtypes

**DOI:** 10.1038/s41598-023-43322-4

**Published:** 2023-10-03

**Authors:** Cyril Malinet, Bruno Montcel, Aurélie Dutour, Iveta Fajnorova, Hervé Liebgott, Pauline Muleki-Seya

**Affiliations:** 1 Université de Lyon, CREATIS, CNRS UMR 5220, Inserm U1044, INSA-Lyon, Université Lyon 1, Lyon, France; 2 Centre de Recherche en Cancérologie de Lyon/Centre Léon Bérard, Equipe mort cellulaire et cancers pédiatriques, UMR INSERM 1052, CNRS 5286, Lyon , France

**Keywords:** Optical spectroscopy, Biomedical engineering, Acoustics, Bone cancer

## Abstract

Histological analysis is the gold standard method for cancer diagnosis. However, it is prone to subjectivity and sampling bias. In response to these limitations, we introduce a quantitative bimodal approach that aims to provide non-invasive guidance towards suspicious regions. Light backscattering spectroscopy and quantitative ultrasound techniques were combined to characterize two different bone tumor types from animal models: chondrosarcomas and osteosarcomas. Two different cell lines were used to induce osteosarcoma growth. Histological analyses were conducted to serve as references. Three ultrasound parameters and intensities of the light reflectance profiles showed significant differences between chondrosarcomas and osteosarcomas at the 5% level. Likewise, variations in the same biomarkers were reported for the two types of osteosarcoma, despite their similar morphology observed in the histological examinations. These observations show the sensitivity of our techniques in probing fine tissue properties. Secondly, the ultrasound spectral-based technique identified the mean size of chondrosarcoma cells and nuclei with relative errors of about 22% and 9% respectively. The optical equivalent technique correctly extracted scatterer size distributions that encompass nuclei and cells for chondrosarcomas and osteosarcomas ($R^2 = 0.80$ and $R^2 = 0.73$ respectively). The optical scattering contributions of nuclei were estimated at 52% for the chondrosarcomas and 69% for the osteosarcomas, probably indicating the abundant and the absent extracellular matrix respectively. Thus, the ultrasound and the optical methods brought complementary parameters. They successfully estimated morphological parameters at the cellular and the nuclear scales, making our bimodal technique promising for tumor characterization.

## Introduction

In clinical settings, the process of cancer characterization involves the determination of the tumor histological subtype and the grade, which reflects the cancer aggressiveness^1^. Determining these characteristics plays a crucial role in establishing the patient's diagnosis and planning the right treatment options. This histological classification relies on cellular morphological measurements and is traditionally determined through microscopic examination, referred to as histological analyses. Although histo-cytopathology serves as a gold standard for diagnosing cancers, this technique is inherently invasive and resource-intensive. Additionally, it is subject to inter-observer and intra-observer variabilities^2^. For instance, the sampled sections may not include the most aggressive cancerous regions due to tumor heterogeneities. Thus, the sampling bias could lead to inaccurate diagnostics that ultimately worsen the patient’s prognosis. Other applications involved in the cancer patient care workflow suffer from subjectivity, such as intra-operative margin assessment^3^. Indeed, insufficient margins resulting in the presence of remaining cancerous cells after resection surgery can lead to cancer recurrence. Consequently, guiding the clinicians toward the most suspicious regions using a non-invasive quantitative tool would be of great benefit for different clinical procedures and could subsequently improve the patient outcomes.

As a starting point toward this objective, two optical and two ultrasound quantitative techniques have been combined on a benchtop to lead to a thorough tissue assessment. The backscatter coefficient (BSC) parametrization and the envelope statistics (ES) are two ultrasound techniques that can extract different tissue scattering properties from the same acquisition^4,5^. Likewise, enhanced backscattering spectroscopy (EBS) and light scattering spectroscopy (LSS) are two light-based techniques that can be performed using a similar experimental setup to characterize biological tissues^6,7^. By combining light and ultrasound, one can expect that the scattering process would arise from cellular structures of varying sizes given the distinct wavelength ranges associated with each modality. Thus, this bimodal technique should have the potential to bring complementary information about the microstructures in the probed tissue.

Unlike conventional ultrasound imaging, which primarily provides anatomical information through B-mode images, quantitative ultrasound techniques aim to provide quantitative measurements that can be used for diagnostic purposes. When analyzing the backscattered radiofrequency (RF) signals used to generate ultrasound scans, valuable information regarding the microstructures of the underlying tissue can be obtained through spectral content analysis (such as the BSC parametrization) or by studying the statistics of its envelope (referred to ES for Envelope Statistics). Successful applications of BSC parametrization and ES provided cancer type classification^8^ and cancerous lymph node characterization^9,10^ for example. BSC inversions can also identify nuclear structures as scatterers in diluted biological media^5^. Similarly, the cell size distribution can also be extracted in dense media^11^.

EBS aims to optically characterize biological tissues. In this method, the tissue characterization is based on the measurements of the angular coherent peak observed in the backscattering direction. This two-dimensional peak then allows the estimation of the spatial reflectance profile, which is the parameter of interest. Numerous studies have investigated the ability of EBS to detect ultrastructural alterations in the field carcinogenesis^12–14^. Radosevich et al.^15^ showed that alterations in the reflectance profiles occurred at short length scales for colorectal and pancreatic cancer field carcinogenesis. EBS can also extract the scattering parameters that shape the phase function to determine the tissue optical properties^13^.

Light scattering spectroscopy (LSS) is another relevant tool that aims to estimate the morphological properties of tissue^16^. It can be performed using an experimental setup similar to the one used for EBS. This technique relies on the spectral analysis of the single scattering component of light and is somewhat the optical equivalent of the BSC parametrization. Backman et al.^17^ successfully extracted the nucleus size distribution from malignant and normal intestinal cells in situ. A few years later, Fang et al.^16^ introduced an analytical procedure to extract the subcellular organelle size distribution without making any inferences about the distribution. Qiu et al.^18^ used this method to show that the nuclear size distributions can be accurately estimated in a dysplastic and in a non-dysplastic site in a Barrett’s esophagus. More recently, Qiu et al.^7^ designed a LSS scanning system able to detect esophageal dysplastic sites with 96% sensitivity and 97% specificity.

Previously, our experimental setups for EBS and BSC parametrization were validated in vitro on three tissue-mimicking phantoms which consisted of suspended microparticles of different sizes^19^. Complementarities in terms of sensitivity to the scatterer sizes were observed with the two techniques. These interesting results led us to carry out this ex vivo animal study to further investigate the performances of a bimodal approach.

In this study, BSC parametrization, ES, LSS and EBS were used to characterize two bone cancer histological types: chondrosarcoma and osteosarcoma established in rodents. The rat chondrosarcoma model reproduces the histological and aggressivity characteristics of grade II human chondrosarcoma. The murine osteosarcoma models used (K7M2 and MOS-J) are representative of conventional metastatic osteosarcoma. Optical and ultrasound measurements were carried out on the day of sacrifices. Quantitative parameters were then estimated and compared between different tumor types. Simultaneously, histological analyses were conducted for all tumors. Morphometric measurements of cellular structures, derived from these examinations, were then compared to evaluate the performances of the BSC parametrization and LSS in estimating scatterer parameters related to cells or nuclei. After analyzing the results, the performances are discussed. Last, the theory and the implementation of each method are exposed.

## Results

### Animal models

**Figure 1:**
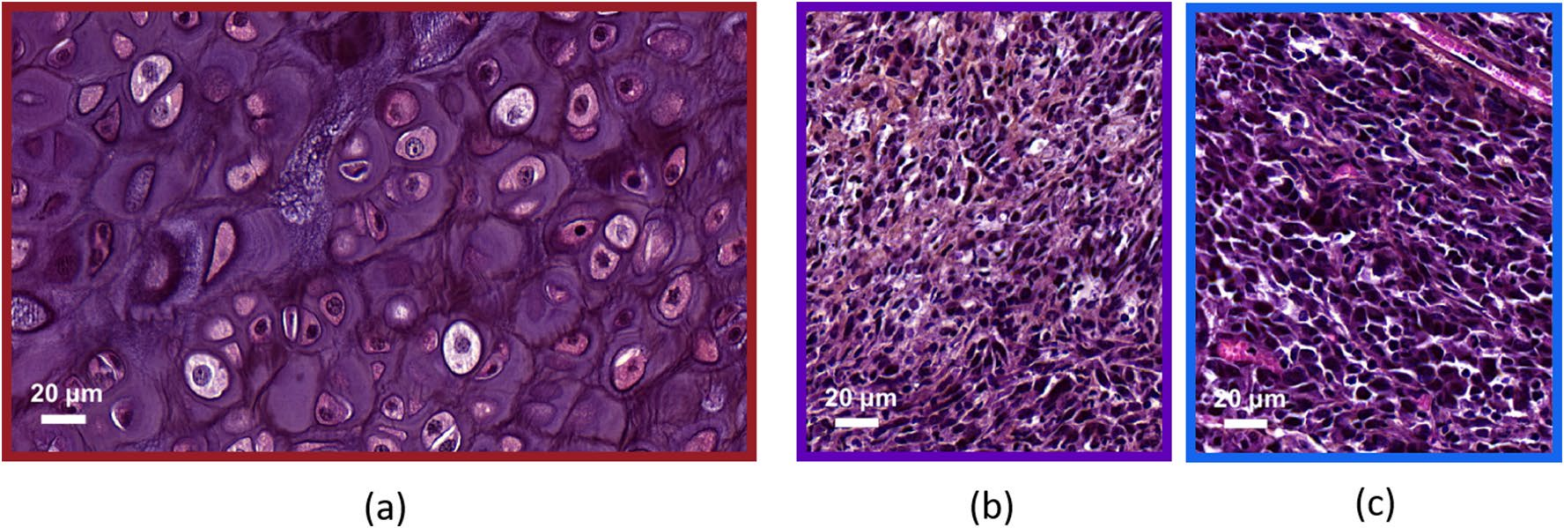
Representative histological stainings of chondrosarcoma and osteosarcoma. (**a**) Chondrosarcoma. HPS staining enables to distinguish cell nuclei, membranes and abundant extracellular matrix. (**b**) and (**c**) Osteosarcomas (K7M2 and MOS-J model respectively). HPS staining shows smaller cells with large nuclei in comparison to chondrosarcoma. Osteosarcomas exhibit a higher cellular density. The absence of extracellular matrix is also observed.

Histological analyses are conducted to serve as references. Morphometric measurements of cellular structures are carried out using the histological slices of tumors to compare the performances of each technique. Chondrosarcoma is characterized by low cell density within an abundant extracellular matrix. One can clearly distinguish extracellular membrane, cell cytoplasm and cell nuclei (Fig. 1a). Both osteosarcoma models are characterized by high cellular density and the absence of extracellular matrix. Osteosarcoma cells exhibit a different morphology in comparison to chondrosarcoma cells. They are smaller in size and have large nuclei (Fig. 1b,c). Hence, histological staining reveals specific morphological features in each bone sarcoma model.

The cell and nucleus size distributions are also estimated using the microphotographs of histological slices.

Chondrosarcoma cells exhibit radii with a mean value and a standard deviation of $9.5 \pm 2.6$ $\upmu$m, while K7M2 osteosarcoma cells have radii of $4.7 \pm 0.9$ $\upmu$m and MOS-J osteosarcoma $4.8 \pm 1.0$ $\upmu$m. Osteosarcoma cells are about twice as small as chondrosarcoma cells. The size distributions of osteosarcoma cells appear more uniform compared to chondrosarcomas. Chondrosarcoma nuclei exhibit radii with a mean value and a standard deviation of $4.2 \pm 0.5$ $\upmu$m, while K7M2 osteosarcoma nuclei have radii of $2.4 \pm 0.6$ $\upmu$m and MOS-J osteosarcomas $2.7 \pm 0.7$ $\upmu$m. Osteosarcoma nuclei appear approximatively twice as small as chondrosarcoma nuclei. The size distributions of chondrosarcoma nuclei are sharper compared to osteosarcomas. Similar cell sizes can be observed between K7M2 and MOS-J osteosarcomas. However, these two osteosarcoma types exhibit slight variations in the nucleus radii.

The chondrosarcoma cell volume fraction is estimated at $\phi _{Ch,cell}=0.23$ and $\phi _{Os,cell}=0.88$ for the two osteosarcoma types, indicating that osteosarcoma is a hypercellular histological subtype that contains almost no extracellular matrix. The nucleus volume fraction is estimated at $\phi _{Ch,nuc}=0.03$ for the chondrosarcoma and $\phi _{Os,nuc}=0.25$ for the osteosarcomas.

### Tumor characterization

#### Quantitative ultrasound

**Figure 2:**
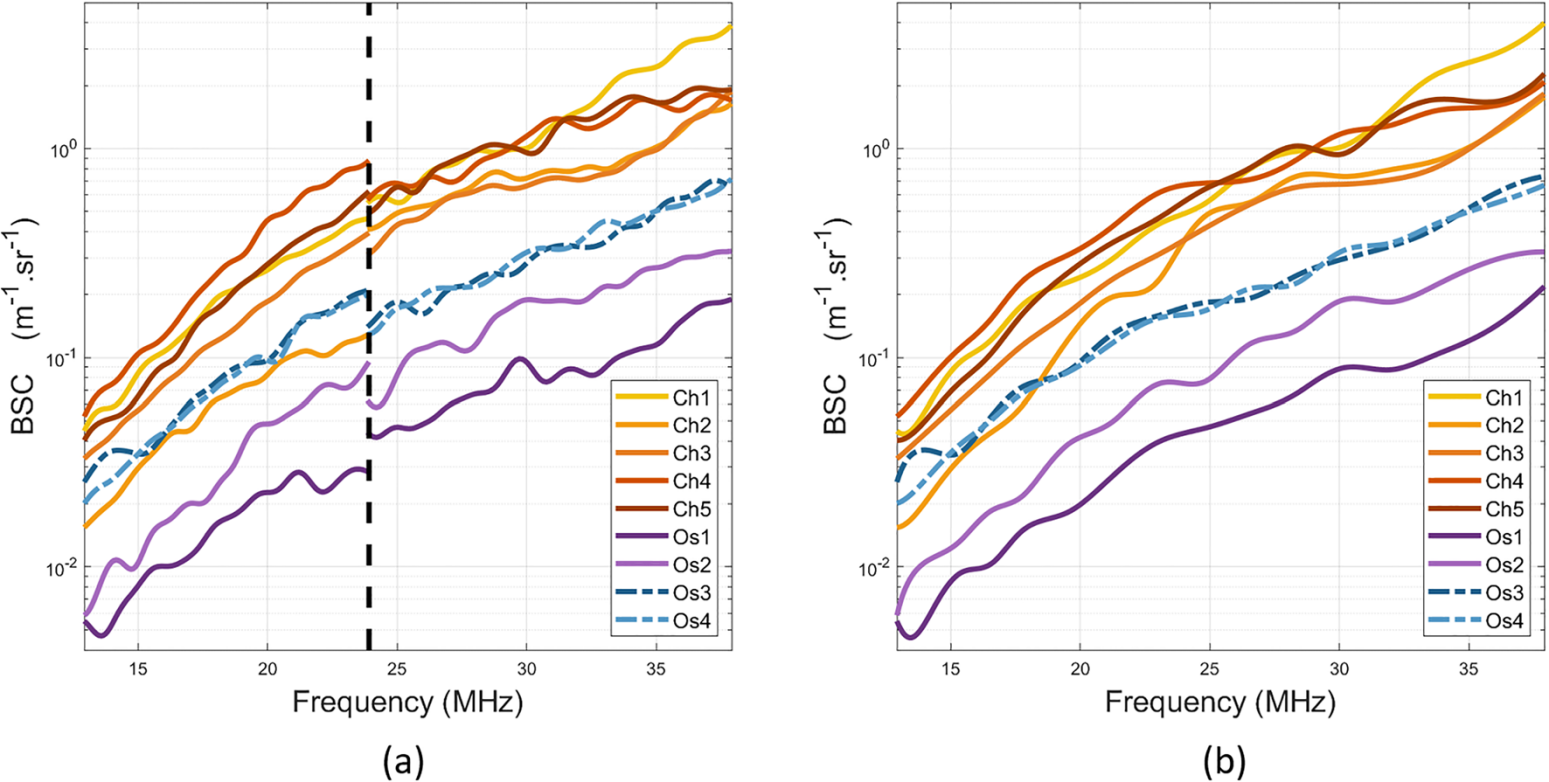
(**a**) Mean estimated backscatter coefficients (BSC) with the MS-250S probe (left of the black dotted line) and the LZ-400 probe (right of the black dotted line) per animal. (**b**) Corresponding BSC b-spline fits. ’Ch’ stands for chondrosarcomas and ’Os’ for osteosarcomas.

Mean BSCs per animal using the low (13–24 MHz) and the high-frequency probes (restricted to 24–38 MHz) are presented in Fig. 2a. The corresponding b-spline fits in the whole frequency range are shown in Fig. 2b. The BSCs exhibit differences between the tumor types and similar trends among tumors of the same nature. The two different osteosarcoma cell lines (MOS-J and K7M2) lead to highly contrasted BSCs.

**Figure 3:**
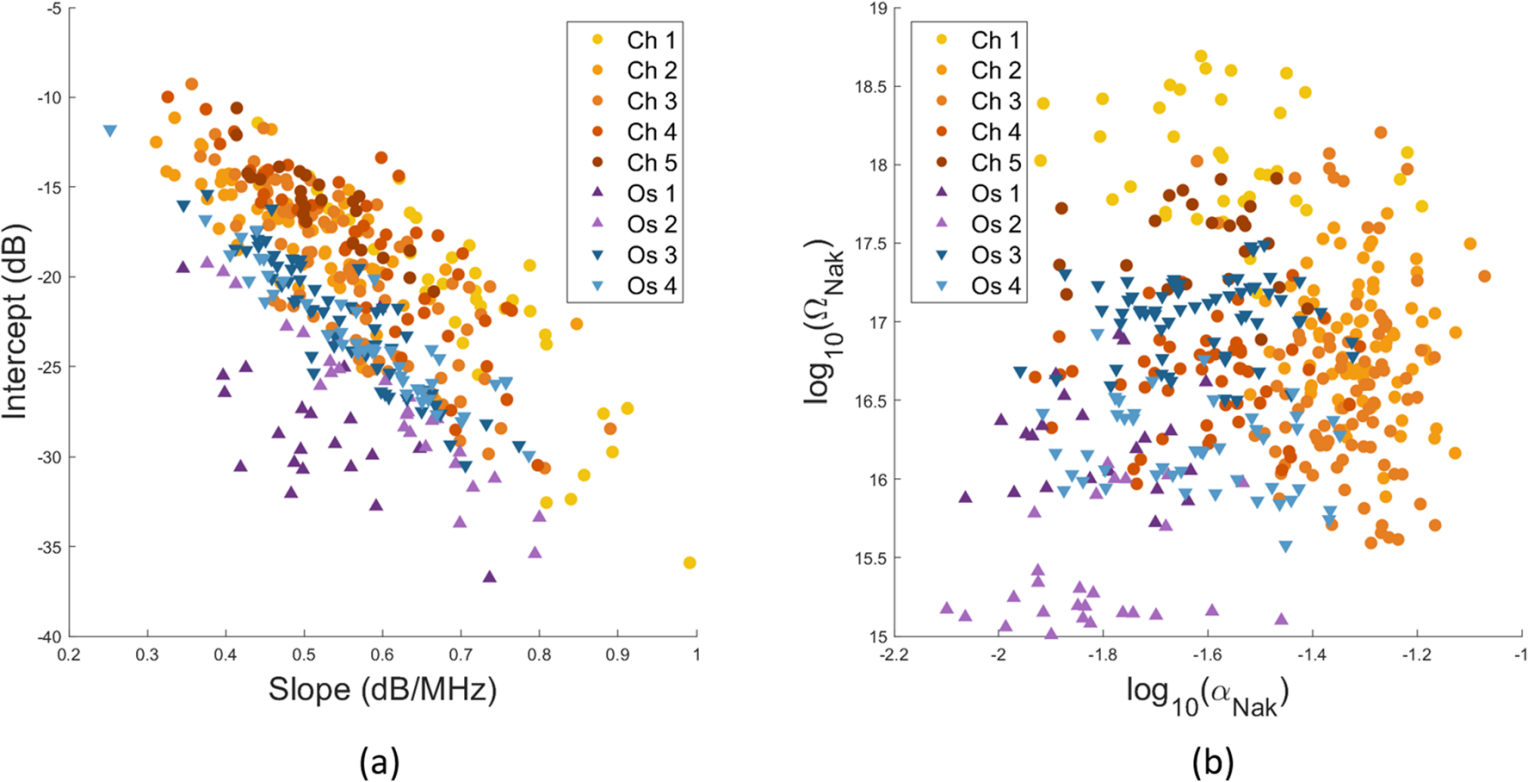
Scatter plots by model. Each point represents an independent region of interest (ROI). (**a**) Intercept versus slope (BSC linear model over the 18–38 MHz frequency range). (**b**) Nakagami envelope model estimated over the 18–38 MHz frequency range. Up and down arrows represent osteosarcomas from MOS-J and K7M2 cell lines respectively.

The differences within the BSCs per ROI are translated into the Lizzi–Feleppa parameters in the scatter plots shown in Fig. 3a. A Wilcoxon rank sum test conducted at a significance level of 5% reveals statistically significant differences in the intercept values between chondrosarcomas and osteosarcomas. However, there is no evidence indicating significant differences in the slope values between the two tumor types (t-test *p*-value = 0.73). The ES parameters are shown in Fig. 3b. The scaling parameters $\Omega$ and the Nakagami parameters $\alpha$ underwent compression using a base-10 logarithm due to their extensive value range. The observed $\alpha$ values indicate Nakagami-gamma distribution ($\alpha < 0.5$)^8^. Wilcoxon rank sum tests report statistically significant differences in the compressed $\alpha$ and $\Omega$ coefficients at the 5% significance level between chondrosarcomas and osteosarcomas.

The same observations can be made for K7M2 and MOS-J osteosarcomas. Student’s t-tests reveal significant differences between the two osteosarcoma types for the intercept values and the Nakagami parameters but not for the slope. The compressed $\Omega$ coefficients also show significant differences in this case. In summary, the three ultrasound parameters (the intercept, $\alpha$ and $\Omega$) can discriminate the chondrosarcomas from the osteosarcomas, and the K7M2 from the MOS-J osteosarcomas. The linear slope appears irrelevant for tumor classification in this study.

#### Light enhanced backscattering spectroscopy

**Figure 4:**
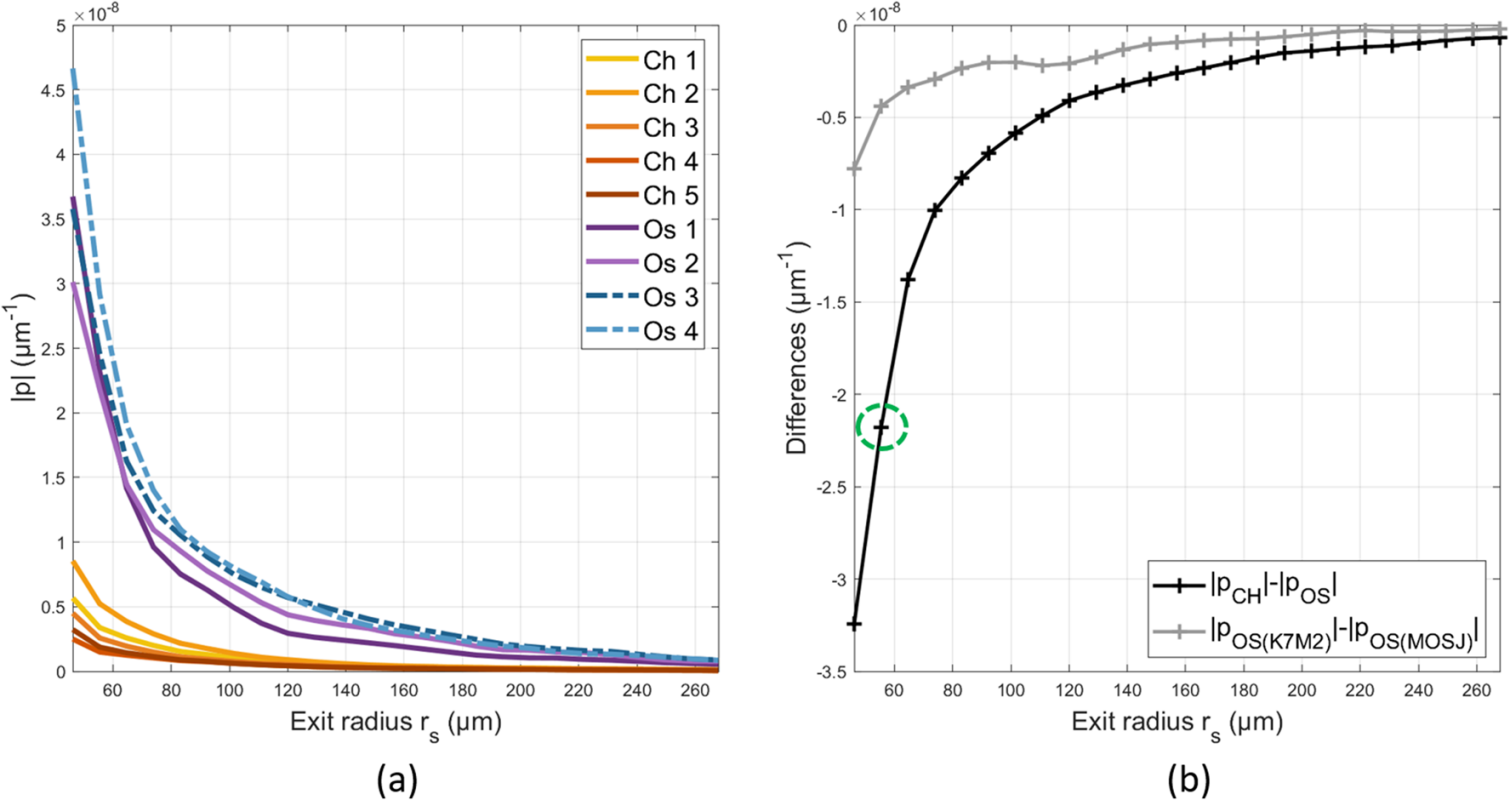
(**a**) Mean reflectance profiles per animal in the co-polarized channel at 700 nm. Statistical significances between chondrosarcomas and osteosarcomas signals were observed using a two-tailed student’s t-test at the 5% level in the whole exit radius range. The location with the most significant changes occurred at $r_{s,opt} = 55$ $\upmu$m with a *p*-value reaching $3\times 10^{-6}$. No significant statistical differences were found at the 5% level between MOS-J and K7M2 osteosarcomas. (**b**) Absolute difference of mean reflectance profiles per group. The green dotted circle indicates the $r_{s,opt}$ of the difference curve.

The reflectance profiles measured in the co-polarized channel at 700 nm are plotted in Fig. 4a. The five chondrosarcoma signals exhibit fast decay and can be clearly identified from the osteosarcomas. This is highlighted by the differences between the mean reflectance profiles of each group, which increases for small exit radius values (Fig. 4b. Statistical significances for each exit radius value were observed using a two-tailed student’s t-test at the 5% level. The location with the most significant changes between chondrosarcomas and osteosarcomas occurred at $r_{s,opt} = 55$ $\upmu$m with a *p*-value reaching $3\times 10^{-6}$. The K7M2 osteosarcomas (Os1 and Os2) signals exhibit a faster decay compared to the MOS-J osteosarcomas (Os3 and Os4). Similarly, differences in mean reflectance profiles between the two types of osteosarcoma increase at small length scales (Fig. 4b) but no significant statistical differences were found at the 5% level.

### Scatterer size distribution

#### Ultrasound BSC parametrization

**Figure 5:**
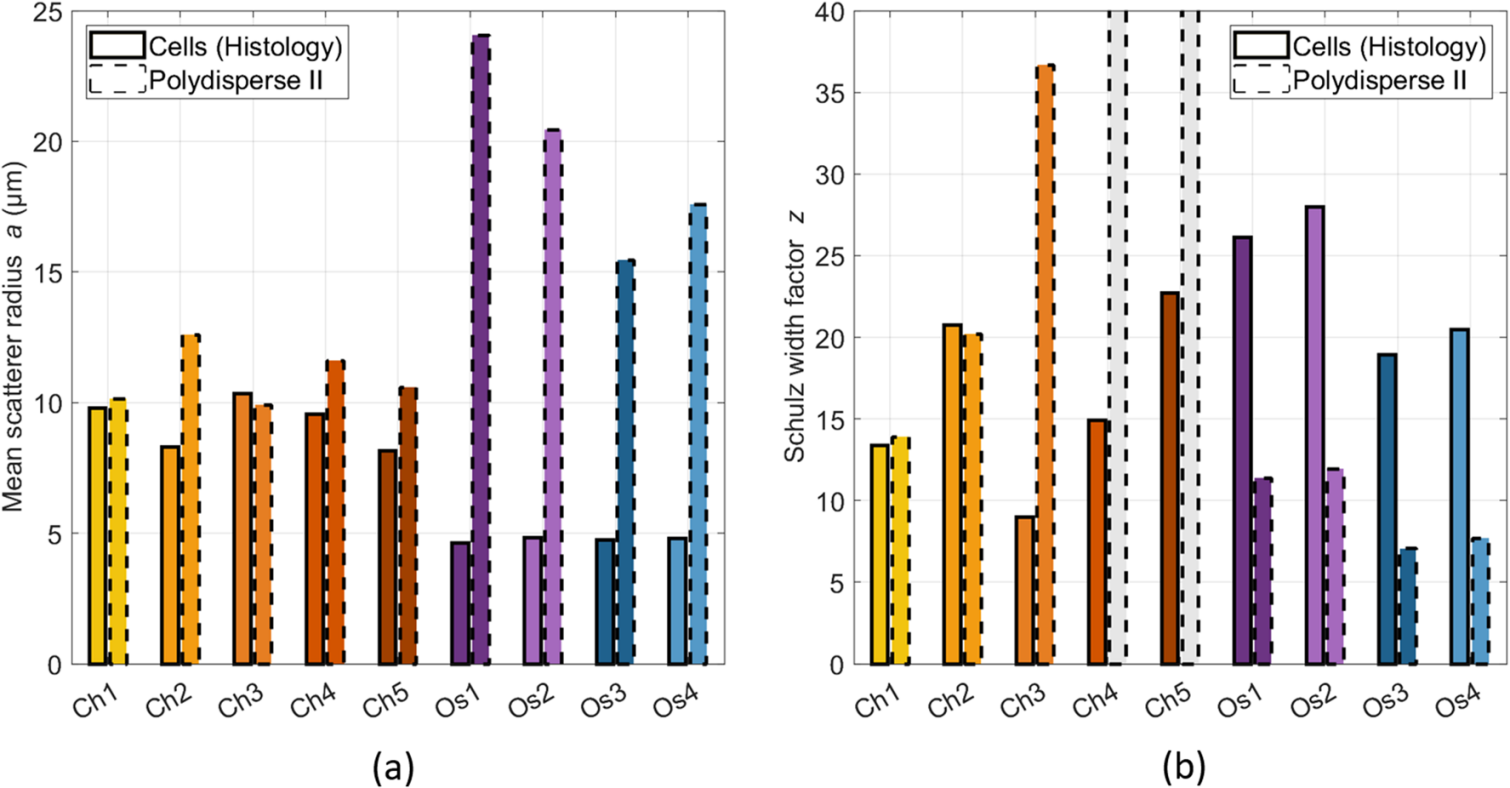
Inversion results using the Polydisperse II model per animal. The cell volume fraction is supposed to be known *a priori* and is set to $\phi _{Ch,cell} = 0.28$ for chondrosarcomas and $\phi _{Os,cell} = 0.88$ for osteosarcomas after histological analyses. (**a**) Mean scatterer radius. (**b**) Schulz width factor. The estimated Schulz width factor for Ch3 and Ch4 reached the upper bound of the inversion constraints.

The BSC inversions using the Polydisperse II (PII) model in the low-frequency range are shown in Fig. 5. The fitting procedure was performed using the average BSCs per animal ($R^2 > 0.99$). The volume fractions were set to the cell volume fraction based on histological estimations. The estimated chondrosarcoma mean scatterer sizes correspond with the mean cell sizes extracted from the histological analyses with a mean relative error of 22% (Fig. 5a). However, these similarities are not observed for the osteosarcomas: the PII model identifies larger scatterers for each tumor (mean relative error> 100%). The Schulz width factors *z* were extracted with relative errors about 3% for Ch1 and Ch2 and superior to 100% for Ch3 (Fig. 5b). The estimated Schulz width factor for other chondrosarcomas reached the upper bound of the inversion constraints. The distribution sharpness is systematically underestimated for the osteosarcomas. The same inversions were conducted by setting the volume fraction to the nuclei volume fractions to validate the results. A minimum mean relative error of 65% was found for all estimates.

**Figure 6:**
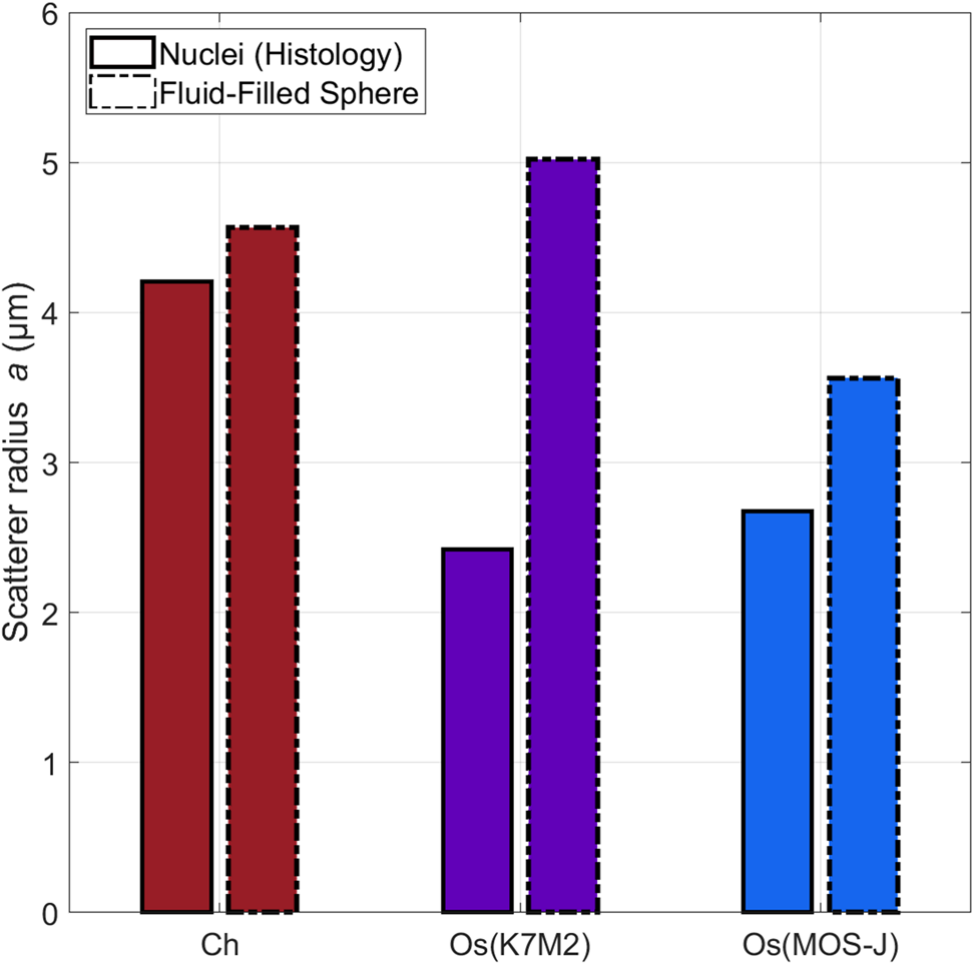
Inversion results using the Fluid-filled sphere model (FFSM) per group in the 24–38 MHz bandwidth. The nucleus volume fraction is supposed to be known *a priori* and is set to $\phi _{Ch,nuc} = 0.03$ for chondrosarcomas and $\phi _{Os,nuc} = 0.25$ for osteosarcomas. The solid bars show the mean nucleus radii estimated by histological analyses.

Figure 6 shows the scatterer radii extracted by the Fluid-Filled sphere model (FFSM) per tumor type in the high-frequency range. The fitting procedure ($R^2 > 0.97$) was performed using the average BSCs per tumor type. In this case, the volume fractions were set to the nucleus volume fraction after histology analyses ($\phi _{Ch,nuc} = 0.03$ and $\phi _{Os,nuc} = 0.25$). The chondrosarcoma scatterer radii estimated by the FFSM correspond to the mean nucleus size extracted in histology with a relative error equal to $9\%$. The osteosarcomas scattering structures identified by the BSC theoretical model are larger than the histological measurements (relative errors > 33%). The PII model estimated the nucleus radius with relative errors equal to 70%, 15% and 49% for the chondrosarcomas, the K7M2 and the MOS-J osteosarcomas respectively. All the estimations of the Schulz width factor reached the lower bound of the inversion constraint using the PII model. Therefore, we did not further consider these inversion results.

**Figure 7:**
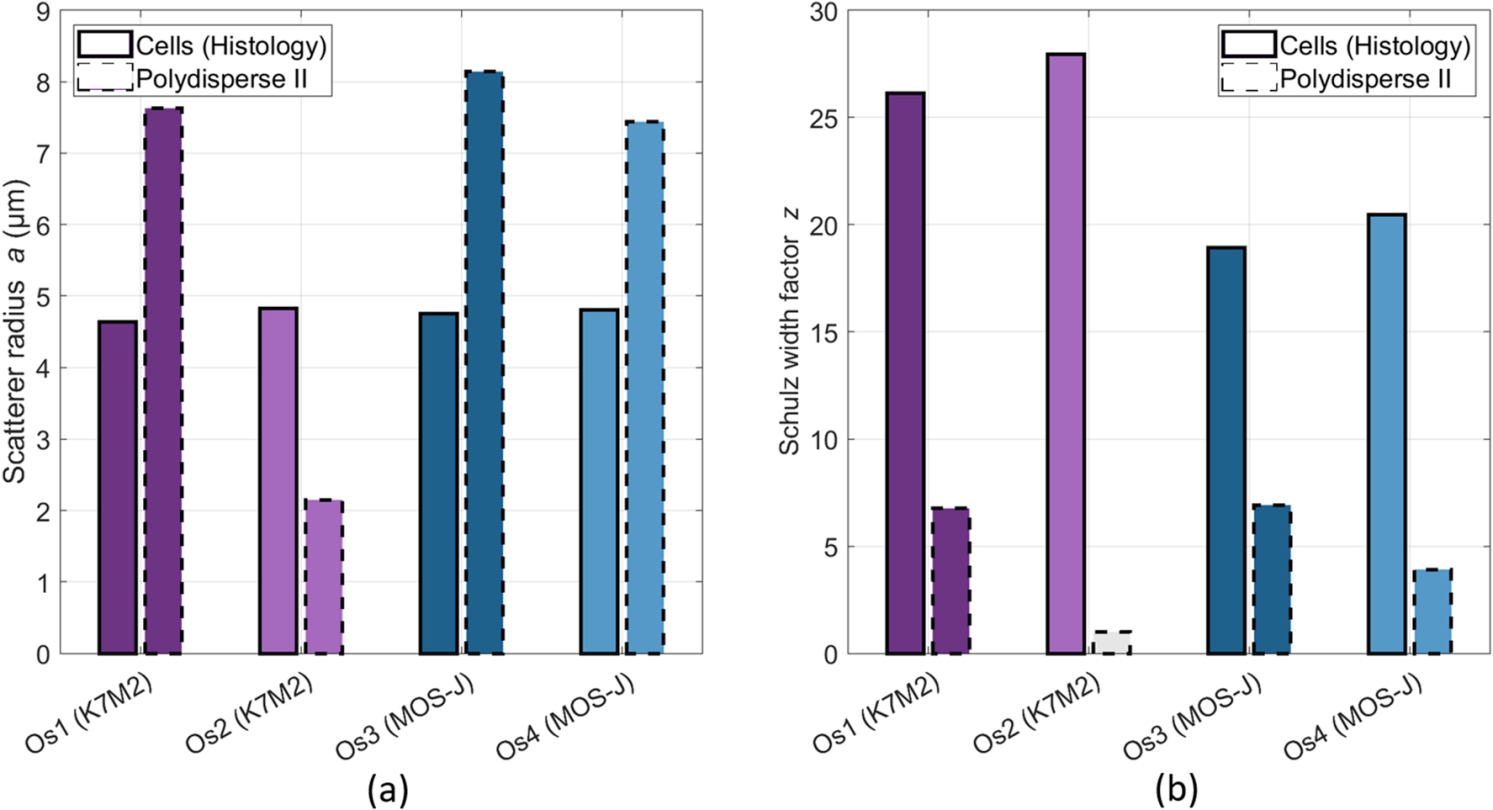
Inversion results using the Polydisperse II model for the osteosarcomas using the BSC b-spline fits (13–38 MHz bandwidth). The cell volume fraction is supposed to be known *a priori* and is set to $\phi _{Os,cell} = 0.88$ for osteosarcomas. (**a**) Mean scatterer radius. (**b**) Schulz width factor. The estimated Schulz width factor for Os2 reached the lower bound of the inversion constraint.

The BSC inversions with the PII model using the BSC b-spline fits (Fig. 2b) for the osteosarcomas are shown in Fig. 7 ($R^2 > 0.97$). Here, the volume fraction was set to the cell volume fraction ($\phi _{Os,cell} = 0.88$). No clear correspondences were observed between the BSC-based parameters and the cell sizes using the whole frequency range (relative errors superior to 50%, Fig. 7a). The Schulz width factors *z* were poorly extracted (relative errors superior to 50%, Fig. 7b). Indeed, the distribution sharpness is underestimated by the PII model. The estimated *z* coefficient for Os2 reached the lower bound of the inversion constraint. The same inversions were conducted by setting the volume fraction to the nuclei volume fractions to validate the results. The osteosarcoma nuclei were estimated with a relative error of 18%. However, all the estimations of the Schulz width factor reached the lower bound of the inversion constraint. Therefore, we did not further consider these results.

#### Light scatttering spectroscopy

**Figure 8:**
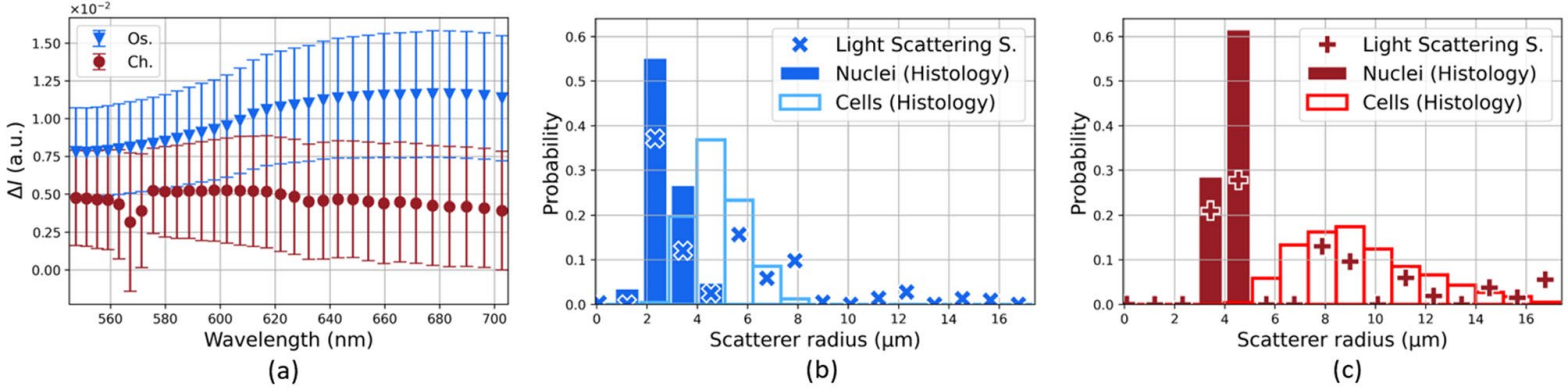
(**a**) Mean differential polarization signal ± standard error for the two tumor types. (**b**) and (**c**) Estimated scatterer size distribution for the osteosarcoma and the chondrosarcoma respectively. The nuclear and cellular size distributions estimated from histological analyses are normalized.

Figure 8a shows the mean differential polarization signals for each tumor type. The measured LSS spectra exhibit significant differences between the two tumor types. The corresponding estimated size distributions *F* are shown in Fig. 8b,c for $r_{max}= 16.75\,$ $\upmu$m. The integral of each distribution is normalized (i.e. cells and nuclei from histological examinations independently analyzed). The shapes of the nucleus size distributions are accurately replicated by LSS. The cell size distributions also appear in the LSS estimations, particularly for the chondrosarcomas.

**Figure 9:**
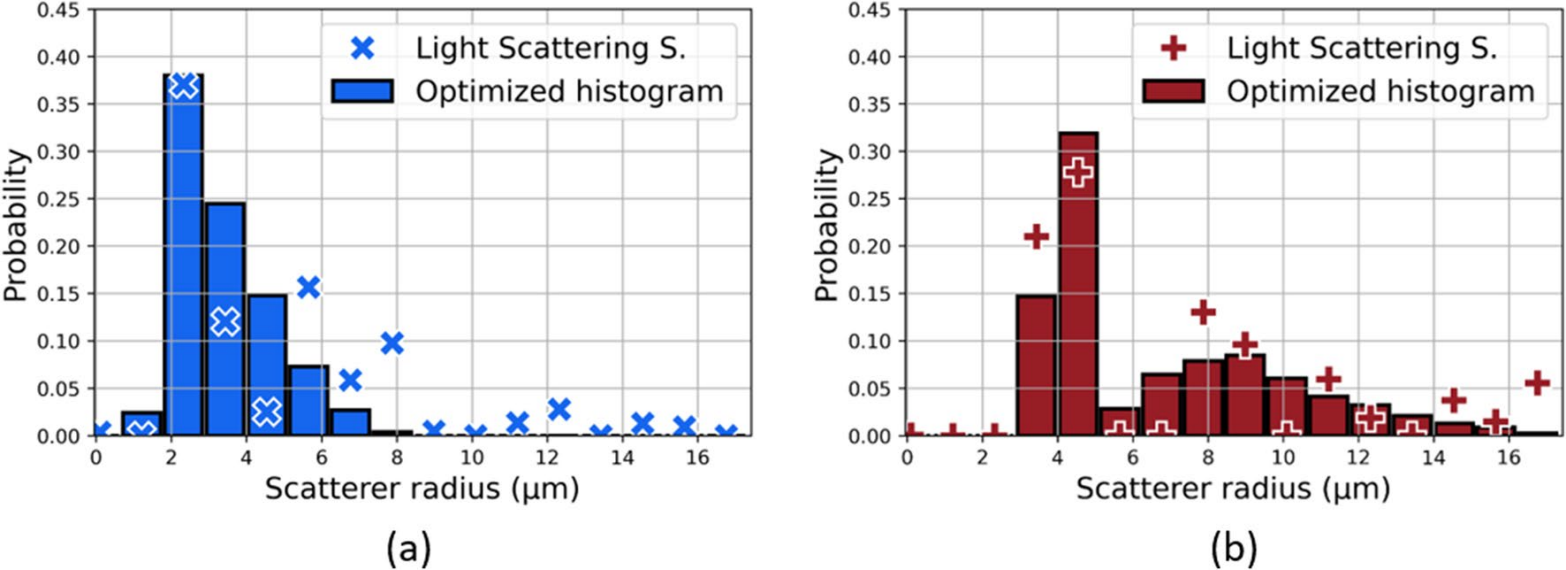
(**a**) Osteosarcoma estimated solution and its optimized linear combination of nucleus and cell size distribution. The nucleus scattering identified in the estimated solution accounts for 69%. (**b**) Chondrosarcoma estimated solution and its optimized linear combination of nucleus and cell size distribution. The cell scattering identified in the estimated solution accounts for 52%.

We hypothesize that cells could be involved in the light scattering process depending on the volume density of the extracellular matrix. In the following procedure, we simply assume that cells and nuclei represent two independent populations of potential scatterers with linearly additive contributions. To test this hypothesis, linear combinations of the nucleus and cell histograms obtained from the histological analyses were computed using different nucleus weight values ($w_{nuc}$). These values were defined such that $w_{nuc} = 1 - w_{cell}$, thus merging the estimated nucleus and cell sizes such as the integral over the scatterer radius equals unity. The optimized nucleus weight was considered as the value that leads to the best fit between the estimated LSS solution *F* and the newly merged histogram. In other words, this procedure redistributes the probabilities obtained with histological analyses to quantify the contribution of nucleus and cell scattering in the LSS solution *F*. Figure 9a,b shows the obtained optimized histograms for each tumor type. The histogram optimization procedure resulted in an estimated nucleus weight of 69% for osteosarcomas and 52% for chondrosarcomas. The coefficient of determination between the estimated size distribution $F_{LSS}$ and the optimized histograms $R_{Ch}^2(F_{LSS},F_{opt,histo})$ equals 0.80 for chondrosarcomas and $R_{Os}^2(F_{LSS},F_{opt,histo})$ equals 0.73 for osteosarcomas.

## Discussion

Two quantitative ultrasound and two light backscattering techniques have been combined to characterize ex vivo tumors. BSC parametrization, ES, and EBS were performed to discriminate each tumor type based on quantitative estimates. Then, BSC parametrization using other theoretical scattering models and LSS were conducted to estimate the scatterer size distribution. Results were compared with histological analyses to study the agreement with cell and nucleus size distributions.

Firstly, significant differences were observed between chondrosarcomas and osteosarcomas in the Nakagami parameters $\alpha$, the scaling parameter $\Omega$, the BSC linear intercept and the light reflectance profile intensity. These results align with the distinct microarchitectures observed in each histological subtype since these scattering parameters reflect the underlying tissue microstructure. Surprisingly, the same three ultrasound parameters show significant variations within the two osteosarcoma cell lines (K7M2 versus MOS-J cell lines). This result was not expected since these cell lines lead to the same tumor model. Indeed, the visual aspects of the histological slices in the microphotographs are not sufficient to identify the specific cell line that induced the osteosarcoma. Moreover, the K7M2 and the MOS-J osteosarcomas exhibit similar volume fractions and cellular sizes. Thus, the observed contrasts between their BSCs could probably be explained by important differences in their relative impedance contrasts $\gamma _z$. This result illustrates the high sensitivity of the BSC parametrization technique to probe fine tissue properties. Likewise, the reflectance profiles showed variations at small exit radii between the osteosarcomas types. However, the limited number of EBS measurements does not provide sufficient evidence to draw conclusions regarding the statistical significance of the observed difference. However, the striking contrasts in EBS signals between chondrosarcomas and osteosarcomas accurately reflect the pronounced differences in their respective microarchitectures. These results are in line with the EBS’s philosophy. EBS is applied in biological tissues to probe submicron microarchitectures by analyzing the reflectance profiles at small length scales relative to the light transport mean free path^15^. Indeed, EBS is used to detect early cancerous cells located in the epithelial layers that are invisible to histological biomarkers. In this study, the extreme sensitivity of this tool is reported with the highly contrasted EBS signals between two completely distinct microarchitectures, as well as the finer differences observed at small length scales for microarchitectures that share a similar histological appearance (K7M2 and MOS-J osteosarcomas).

Secondly, BSC inversions were conducted to investigate the extent to which cellular structures could be regarded as ultrasound scatterers in chondrosarcomas. The PII model (Fig. 5a) successfully identified the mean chondrosarcoma cell size and two Schulz width factors out of five estimated the sharpness of the cell size distribution in the low-frequency range. However, poor correspondences between the Schulz parameters and the histological analyses were observed in other cases, leading to mainly unstable estimations of this parameter. Interestingly, Han et al.^11^ reported higher relative errors for the estimations of the Schulz width factor compared to the mean scatterer radius using the PII model in cell pellet biophantoms. Indeed, simulations revealed that the BSC shape is more sensitive regarding the mean radius than the Schulz parameter z in the PII model (data not shown). Thus, the estimation of z is more subject to experimental noise, thereby increasing the difficulty of its accurate determination. The mean nucleus radii of the chondrosarcomas were correctly estimated by the FFSM model in the high-frequency range at the expense of the averaging process of all the independent BSC estimations. Conversely, a limited number of independent LSS measurements were sufficient to accurately extract the nucleus size distribution. However, the LSS estimation of the cell size distribution was less precise but allowed to quantify the contribution of cell scattering in the observed spectrum. Indeed, approximately half of the chondrosarcoma LSS spectrum can be attributed to cell optical scattering, while the remaining half corresponds to nucleus scattering. These results are coherent with the histological analyses and the simple microarchitecture observed in this tumor type. Indeed, chondrosarcoma tumors are characterized by a low cell density. Moreover, chondrosarcoma cells and nuclei exhibit limited size overlapping, thereby facilitating the clear identification of each structure. Thus, we make the hypothesis that the cells could be considered as discrete optical scatterers surrounded by the abundant extracellular matrix, similar to how nuclei are usually modeled as isolated scatterers surrounded by cytoplasm.

The same protocol was carried out for the osteosarcomas. The BSC parametrization systematically overestimated the osteosarcoma cell radius. To explain this result, we postulated that the center frequency may not be sufficiently high to induce scattering from the osteosarcoma cells, which are smaller than the chondrosarcoma cells. This led us to carry out another inversion procedure using the b-spline BSCs (Fig. 7b) by setting the volume fraction to the cell volume fraction estimated after histology analyses. The PII model did not identify the cells as scatterers either. Besides, the BSC parametrization successfully identified the chondrosarcoma nuclei, which are approximately as small as the osteosarcoma cells. Thus the insonification frequency was not believed to be too low for osteosarcoma cells. These observations brought us to formulate a second hypothesis which is that the scattering from osteosarcoma cells may not be predominant. The LSS size distribution estimation led us to the same observation and attributed less than 30% to optical cell scattering in the measured spectra. We suggest that these results arise from the hypercellular nature of osteosarcoma tumors. Indeed, this tumor type contains almost no extracellular matrix (Fig. 1b) and presents contiguous cells. Thus, competing ultrasound and optical scattering from other structures may potentially mask the scattering signals from cells. Moreover, the significant size overlap among osteosarcoma cells and nuclei further complicates their discrimination, presenting an additional challenge. One should note that the effects of a high concentration of scatterer per unit volume are taken into account in the ultrasound scattering model PII (Eq. 4). Thereby, structural effects are not sufficient either to explain the failure of BSC parametrization in estimating the osteosarcoma cell sizes.

LSS and BSC inversions were performed to study the degree to which nuclei could be considered as scatterers in the osteosarcomas. LSS successfully extracted the nucleus size distribution and outperformed the FFSM inversion results, which overestimated the nucleus size. This may be explained by the fact that the ultrasound frequency was not high enough for the incident wave to interact with the osteosarcoma nuclei. Indeed, the histological analyses show that they are smaller than the chondrosarcoma nuclei. As a result, the products of wavenumber by scatterer radius are equal to $ka_{Os} = 0.39$ and $ka_{Ch} = 0.65$ at 38 MHz.

In summary, the BSC parametrization and EBS appear as relevant tools for discriminating tumor types. Moreover, these techniques detected signal contrasts even among samples that present similar cellular morphologies. Thus, they might provide biomarkers that are invisible to conventional histological diagnostic markers. To estimate the microstructure sizes, the BSC parametrization was complementary to LSS for the study of chondrosarcomas. The first technique was more accurate in the estimation of the mean cell sizes while the second method led to a more efficient extraction of the nucleus size distribution. We argue that these results arise from the correspondences between the simple microarchitectural structures of the chondrosarcoma and the basic geometries assumed in the scattering models. Conversely, identifying the cell size in highly cellular media such as osteosarcoma tumors appears more challenging due to their geometrical cell contiguity and the competing scattering from other microstructures. However, in both cases, LSS can provide valuable insights into the cell size distributions and can quantify the scattering contributions of each object.

Thirdly, potential limitations associated with the present study can be discussed. Light scattering spectroscopy correctly estimated the nucleus size distribution for both tumor types using a certain value of the maximum scatterer radius allowed in the inversion procedure. The numerical stability of the previous solution was investigated by varying the maximum scatterer radius values. The osteosarcoma solutions appeared relatively more robust than the chondrosarcoma solutions. Indeed, any $r_{max}$ values taken within the interval $[16.50\,$ $\upmu$m $,17\,$ $\upmu$m] lead to satisfying and reproducible estimations of the osteosarcoma nucleus size distribution. The observed amplitude of this interval is ten times smaller for the chondrosarcoma. To understand the observed instability of the chondrosarcoma solution, the LSS spectra shape could be analyzed (Fig. 8a). The osteosarcoma spectra appear smoother than the chondrosarcoma spectra, which show a brutal variation around 570 nm. To mitigate the influence of Rayleigh scattering, the LSS spectra are multiplied by $\lambda ^4$ prior to differentiation with respect to $\lambda$. Consequently, the experimental noise in the LSS spectra gets strongly amplified in the signal processing required to estimate the scatterer size distribution. This could explain the poor stability observed for the chondrosarcomas. In brief, the LSS analytical procedure initially described by Fang et al.^16^ appears useful to mitigate Rayleigh scattering and to justify the $r_{min}$ value. However, it also introduces a significant increase in experimental noise, particularly at high wavelengths. The precise estimation of the nuclear size distribution using LSS is challenging, and we argue that this tool should be more robust for classification applications. Indeed, the measured LSS spectra exhibit significant variations (Fig. 8a). This observation aligns with findings from the latest studies. Recent papers investigated the use of a diagnostic parameter based on the differences between LSS spectra from normal and dysplastic sites^7,20^ to detect precancerous lesions. This simple approach led them to outperform the specificity and the sensitivity of recently commercialized optical tools^7^.

Finally, the points discussed above brought us directions for future investigations. The contribution of optical scattering by cells and nuclei brought by LSS covers a great potential that deserves further consideration. As observed in this study, the cell scattering percentage may reflect the volume fraction of the extracellular matrix within the tumor. Consequently, LSS has the capacity to not only provide size measurements but also to estimate the cell density. Considering that cellularity is of prime interest to pathologists, this additional capability enhances the value of LSS in diagnostic applications. Moreover, an estimation of the cell volume fraction by LSS would be of great benefit in BSC fitting procedures. Indeed, ultrasound scattering models are parameterized by multiple independent coefficients that can include the volume fractions. In the fitting procedures conducted here, the volume fractions were set to a fixed value considered to be known *a priori*. Using one LSS output as an input for the BSC parametrization could avoid this hypothesis. Hence, this optical method can be beneficial for the spectral-based ultrasound technique, additionally to providing complementary information. This makes our approach a promising bimodal application for tumor characterization. As opposed to discrete scatterers, biological samples can be considered as continuous random media to encompass their complexity. Under this assumption, the three-parameter Whittle–Matérn function can describe the refractive index correlation function^21^. It is possible to take the EBS analysis one step further and to extract refractive index-related parameters as described in Radosevich et al.^13^. This approach has the potential to provide robust tumor characterizations without making assumptions on the scatterer geometries. For the ultrasound measurements, insonification at higher frequencies could induce scattering from small structures such as the osteosarcoma nuclei, making them potentially detectable through inversion procedures. Future studies will investigate these points.

In conclusion, the two quantitative ultrasound and the two optical techniques brought complementary parameters that reflect the underlying tissue microstructure for different tumor types. The estimated morphological parameters were found to be sensitive to the cellular and nuclear scales. These promising findings lead us to conduct an ex vivo animal longitudinal study to assess the sensitivity of this bimodal technique for treatment monitoring applications.

## Materials and methods

### Animal models

In this study, the use of chondrosarcoma and osteosarcoma tumors is motivated by their different microstructures. Characterizing these tumors appears as a way to validate our bimodal method with the aim of establishing a proof-of-concept. Given that the inner mechanisms of our methods probe the tissue microstructure, our approach could potentially be applied to other types of tumors and to healthy tissues.

The experiment was approved by the ethical committee CECCAP (Comité d’éthique en expérimentation animale de la Région Rhône-Alpes, registration number C2EA15, Lyon, France) and by the the ethical committee ACCESS (Comité d’éthique en expérimentation animale commun Centre Léon Bérard - Centre de Recherche en cancérologie de Lyon, CE010, MESR number: #35086). All methods were conducted in agreement with the established guidelines and with the European and French regulations. This study is reported in accordance with ARRIVE guidelines. For all surgical procedures, pre-analgesia was induced by a subcutaneous injection of buprenorphine (0.05 mg/kg) (ECUPHAR, Belgique). All tumor implantations were performed on anesthetized animals (isoflurane/oxygen, 2.5%/1.5%, v/v) (Minerve, Esternay, France). Five chondrosarcomas tumors, hereafter referred to as Ch1–Ch5, were grafted on 25-d-old Sprague–Dawley rats. Tumor fragments (10 mm^3^) were transplanted on the right posterior tibia of the rats after periostal abrasion^22^. The osteosarcoma models were established by injection of $1\times 10^6$ MOS-J (Os1 and Os2) or K7M2 (Os3 and Os4) suspended cells^23^. Tumor progression was monitored twice a week by palpation and caliper measurements until it reached a 500–600 mm^3^ volume for all tumors. The animals were then euthanized by CO_2_ inhalation and the tumors were removed for optical and ultrasound imaging, which were performed the same day. Then, the tumors were embedded in formalin-fixed paraffin-embedded blocks before undergoing histological analyses. Tumor slices were H &E and Ki67 stained through an automated procedure and scanned to obtain microphotographs. The histological parameters were analyzed using Qupath (software version 0.3.2) to estimate the size distributions of osteosarcoma cells and all nuclei. Osteosarcoma microstructures were measured using H &E images, while chondrosarcoma nucleus sizes were evaluated using Ki67 images. Following segmentation with the automatic detection tool, the radii of cells and nuclei were extracted by assuming the circularity of the detected objects. For chondrosarcoma cells, MATLAB (software version R2020b) and H &E microphotographs were employed to detect cells within bounding boxes. The sizes of the bounding boxes were halved to obtain a characteristic size, considered as the radius. The cell size distribution for each animal was then fitted to a $\Gamma$-distribution to extract the mean radius *a* and the Schulz width factor *z*. As an approximation, the volume fractions of both the cell and nucleus were assumed equal to the surface fractions^24^. The surface fraction represents the ratio of the mean intercepted areas of the object of interest to the total surface area analyzed. An average volume fraction was considered for each tumor type. Only Ch3 and Ch4 contributed to the estimated volume fraction for chondrosarcomas.

### Quantitative ultrasound

#### Ultrasound backscatter coefficient parametrization

The Backscatter Coefficient *BSC* represents the tissue’s ability to backscatter the acoustic energy as a function of the frequency *f*. The BSC parametrization entails in extracting scattering parameters (e.g. the scatterer radius *a*) from the BSC measurements. To achieve this, analytical models are fitted to the BSCs through inversion procedures. A first approach involves fitting the BSC expressed in dB as a linear function. This simple procedure leads to ultrasound parameters known as the ’Lizzi–Feleppa’ coefficients: the intercept, the slope and the midband value. As the midband value is not independent of the other coefficients, only the slope and the intercept were analyzed in this study. More sophisticated models are also used for tissue characterization. Indeed, in biological media considered as sparse (i.e. relatively few scatterers per unit volume), the theoretical BSC can be expressed as the product of the BSC in the Rayleigh limit and the Fluid-Filled Sphere form factor *FF* as follows:

1 $$\begin{aligned} \begin{alignedat}{2} BSC_{in}(k) = n\int _{0}^{\infty }\frac{k^4V_s(x)^2\gamma _z^2}{4\pi ^2}FF(k,x)N(x)dx \end{alignedat} \end{aligned}$$

where *k* is the wavenumber, *n* the scatterer number density, *x* the sphere radius, $V_s(x) = \frac{4}{3}\pi x^3$ the scatterer volume, $\gamma _z = \frac{z_0-z}{z}$ the relative impedance contrast between scatterers and the surrounding medium, *N* the probability density function. In the case of monodisperse scatterers, the integral in Eq. (1) gets simplified and the BSC is then expressed as a function of the following parameters of interest: the volume fraction $\phi =nV_s$, the scatterer radius *a* ($x = a$ in this case) and $\gamma _z$. Hereafter, the Fluid-Filled Sphere model (FFSM) will refer to the following analytical expression:

2 $$\begin{aligned} BSC_{FFSM}(k) = \frac{3\phi ak^2\gamma _z^2}{4\pi }j_1^2(2ka) \end{aligned}$$

where $j_1$ is the spherical Bessel function of the first kind of order 1. However, the previous BSC expressions do not cover the case of dense media. Indeed, it can be assumed that the scatterer position correlation increases with their concentration^11^. When the scatterers are not randomly spatially distributed, structural effects affect the ultrasound backscattering and the BSC is no longer the incoherent sum of the contributions of each scatterer. To take this concentration effect into account, the incoherent signal $BSC_{in}$ can be modulated by a structure factor *S*. The analytical expression of *S*(*k*) for polydisperse scatterers (Polydisperse II model) can be found in Han et al.^11^. In the Polydisperse II (PII) model, the scatterer size distribution is assumed to follow a $\Gamma$-distribution :

3 $$\begin{aligned} N_z(x) = \frac{1}{z!}\left( \frac{z+1}{a}\right) ^{z+1}x^ze^{-(z+1)\frac{x}{a}} \end{aligned}$$

where $N_z$ is the probability density function, *a* the mean scatterer radius, *z* the Schulz width factor (the higher z is, the narrower the distribution). In summary, Han et al.^11^ provided an expression to model the ultrasound backscattering of polydisperse scatterers in concentrated media based on a Fluid-Filled sphere form factor. Under these assumptions, the BSC is a function of the following parameters of interest: the scatterer mean radius *a*, the Schulz width factor *z*, the volume fraction $\phi$ and the relative impedance contrast $\gamma _z$. Hereafter, the PII model will refer to this BSC model:

4 $$\begin{aligned} BSC_{PII}(k) = BSC_{in}(k)S_{PII}(k) \end{aligned}$$

#### Ultrasound envelope statistics

While the BSC parametrization extracts spectral-based parameters, envelope statistics (ES) entails in estimating the attributes of the envelope statistical distribution of the backscattered signals. This procedure leads to additional scattering parameters. The Probability Density Function (PDF) of the measured envelope can be fitted with a Nakagami distribution model to extract the scaling factor $\Omega$ and the Nakagami parameter $\alpha$. The scaling factor $\Omega$ is equal to the mean backscattered intensity^25^ and $\alpha$ can be used to quantify the effective number of scatterers per resolution cell^9^. If *A* is a random variable that follows a Nakagami distribution, then:

5 $$\begin{aligned} \Omega= & {} E[A^2] \end{aligned}$$

6 $$\begin{aligned} \alpha= & {} \frac{E^2[A^2]}{Var[A^2]} \end{aligned}$$

#### Implementation of QUS techniques

The sample was insonified with focused waves using two linear probes (MS250S, LZ400, Vevo LAZR scanner, Fujifilm VisualSonics) centered at 21 MHz and 30 MHz, allowing tissue characterizations over the 13–24 MHz and 18–38 MHz frequency ranges respectively. The use of a high-frequency probe makes the successful characterization of small objects (i.e. nuclei) more likely since the Mie scattering region is targeted ($ka \sim 1$). A 3D scan was performed and consisted of 10 B-mode images spaced out 0.1 mm away from each other with three focals located at 6, 8 and 10 mm for osteosarcomas and 10, 12 and 14 mm for chondrosarcomas. Each scan was composed of 1536 RF lines and imaged the tumor over 15 mm in the lateral direction. Regions of Interest (ROI) were 15$\lambda$ long in both directions and were located at a relatively shallow depth (1–1.5 mm). Our reference phantom was composed of polyamide particles of diameter 5 $\upmu$m (Orgasol 2001 UD, Arkema) at the relative mass concentration of 0.25% in a gel that contains agarose (2%, Sigma) and water. The number of independent ROIs used or averaged for each technique is shown in Table 1. The sample attenuation was estimated using a standard substitution method^26^. The BSC for each ROI was estimated using the reference phantom method^27^. Then, the BSC estimations from each frame were averaged for the MS250S probe and the LZ400 probe (Fig. 2a). A b-spline fit was then performed to merge the BSC estimations from the two probes^11^. When applying a linear model to the measured BSCs, linear fits were filtered out based on their resulting Pearson correlation coefficient using a threshold value $R^2_{min} = 0.60$. This procedure removed less than 5% of the collected data. The inversion procedure was performed using the Matlab function *fminsearchbnd* by minimizing the squared error between the experimental data and the expected model with the following constraints: $(a,z) \in [0.1\,$ $\upmu$m $,100\,$ $\upmu$m$]\times [1,120]$. Multiple seed values for *a* and *z* were tested. The volume fraction $\phi$ was either set to the nucleus or the cell volume fractions.

The scaling parameters $\Omega$ from the Nakagami distribution were obtained using a maximum-likelihood estimator. The estimates $\Omega$ can be corrected for attenuation and diffraction effects as suggested in Mamou et al.^9^ (Eqs. 9 and 10). The correction then allows the comparisons between the ultrasound-based parameters from ROI located at different positions. In this study, envelope parameters have been extracted from the RF data acquired in the 18–38 MHz range.

**Figure 10:**
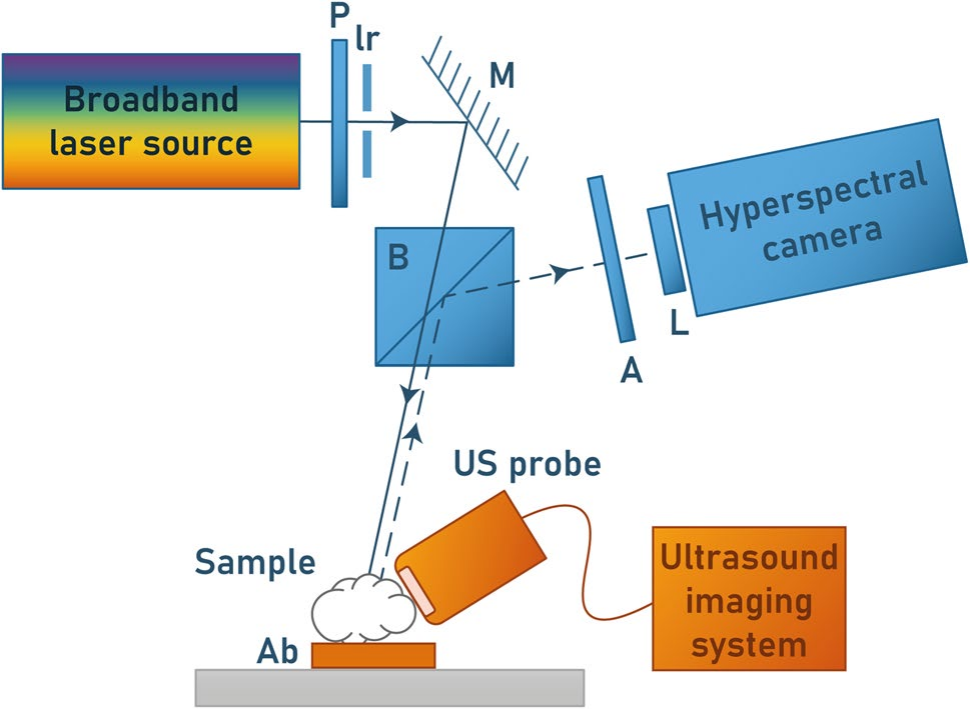
Experimental setup; P: polarizer, Ir: iris diaphragm, M: mirror, B: beamsplitter, A: analyzer, L: Fourier lens, Ab: absorbing material. The detection block was substituted by a filter wheel and a monochrome camera for EBS.

### Light enhanced backscattering spectroscopy

#### Theory

Enhanced backscattering spectroscopy (EBS) entails in extracting the sample spatially resolved diffuse reflectance $p(r_s)$, $r_s$ being the relative spatial separation between the entrance and exit point of multiply-scattered photons in the sample. This quantity is also known as the radial point spread function. This reflectance profile can be seen as an optical tissue signature. Indeed, $p(r_s)$ is extremely sensitive to the phase function in the subdiffusion regime ($r<l_s^*$, $l_s^*$ being the transport mean free path)^6,13^. This can be explained by the fact that the photons that correspond to this regime have undergone few scattering events.

Under the approximation of a semi-infinite medium irradiated by light plane waves, the reflectance profile and the EBS peak are linked by the Fourier transform. The EBS peak is a 2D angular intensity peak in the exact backscattering direction. It results from constructive interferences between all the time-reversed path-pairs photons. Experimentally, a CCD camera can detect this angular intensity distribution. An inverse Fourier transform then gives the effective reflectance profile $p_{eff}(r_s)$ which represents the modulation of $p(r_s)$ by other functions^14^:

7 $$\begin{aligned} I_{EBS}(\theta _x,\theta _y) = FT\{p_{eff}(x_s,y_s)\} = FT\{p(x_s,y_s)\cdot pc(x_s,y_s)\cdot s(x_s,y_s)\cdot c(x_s,y_s)\cdot mtf(x_s,y_s)\} \end{aligned}$$

where *FT* denotes the 2D Fourier transform, $x_s$ and $y_s$ the Cartesian coordinates associated with $r_s$, *pc* the phase correlation function which represents the ability of forward and reversed photons to interfere, *s* a modulation due to finite illumination spot size, *c* the spatial coherence function and *mtf* the imaging system’s modulation transfer function.

#### Implementation

Figure 10 illustrates the experimental setup used for EBS and LSS. The description of the EBS experimental setup can be found here^28^. Briefly, a broadband laser source emits a collimated beam that is directed towards the tumor. The visible power (400–850 nm) was set to 25 mW. The beam is shaped into a circular spot with a diameter of 1.8 mm using an iris diaphragm, ensuring compliance with the Nyquist sampling criterion as described in Radosevich et al.^6^. The non-polarizing beamsplitter has a 50:50 ratio. The tissue sample is immersed in an aqueous solution of glycerol, which has a refractive index similar to that of the assumed tissue refractive index (n = 1.38). To minimize the presence of speckle noise, a motor is used to rotate the sample gently. The analyzer is parallel to the polarizer to select the co-polarized channel. A Fourier lens (focal distance of 50 mm) focuses the light onto a CCD camera. The camera (Thorlabs 340M, not depicted in the diagram) detects the backscattered light filtered at a wavelength of 700 nm (filter FWHM of 10 nm). The camera pixels have dimensions of $7.4\times 7.4$ $\upmu$m. This configuration allows an angular resolution of $8\times 10^{-3}$ °. The outlines of the data processing steps suggested by Radosevich et al.^14^ were followed. In a few words, the sample image was background-substracted and normalized by the total unpolarized incoherent intensity measured from a reflectance standard (SRT-99-050, Labsphere). Then, the incoherent baseline was estimated from a plane fit using data from an annular ring spanning from 0.9° to 1° away from the maximum peak intensity. Then, the incoherent baseline was subtracted from the sample image. To mitigate the effect of unwanted systematic reflection in a half plane, the sample images were mirrored with respect to the horizontal axis that passes through the intensity peak. The rotational averages of the 2D Fourier transform images led to the effective reflectance profiles. The use of a laser and the co-polarized channel enables us to consider *pc* and $c \approx 1$ (Eq. 7). Calibration measurements finally allow the extraction of the sample reflectance profiles from the effective reflectance profiles by estimating the product of *mtf* and *s*. The exit radii were restricted to the range where the reflectance profiles were above the noise level (i.e. $p(r_s)$ values for $r_s$ close to the iris diameter^29^). Tumor size allowed us to perform five EBS measurements corresponding to different positions for the chondrosarcoma tumors and one EBS measurement for the osteosarcomas (Table 1).

### Light scattering spectroscopy

#### Theory

Light Scattering Spectroscopy (LSS) aims to analyze the elastically single scattered photons and can be used to extract the scatterer size distribution *F*. The size distribution is estimated from the sample differential polarization signals $\Delta I$, which isolate the single scattering component. This quantity is somewhat the optical equivalent of the *BSC* mentioned above. LSS models the detected spectrum as the incoherent sum of the contributions of each scatterer^7^.

8 $$\begin{aligned} \Delta I(\lambda ) = \int _{r_{min}}^{r_{max}} {\tilde{I}}(\lambda ,r,n_{re})F(r)\,dr + \frac{C_R}{\lambda ^4}+\epsilon (\lambda ) \end{aligned}$$

where $\lambda$ is the wavelength, $n_{re}$ the relative refractive index between the scatterer and the surrounding medium, ${\tilde{I}}(\lambda ,r,n_{re})$ the LSS spectrum of a single scatterer of radius *r*, $r_{min}$ the radius threshold below which Rayleigh scattering is considered as dominant (typically $100 \,$ nm), $r_{max}$ the maximum scatterer radius, *F*(*r*) the scatterer size distribution, $C_R$ an unknown constant proportional to the number of Rayleigh scatterers and $\epsilon (\lambda )$ the experimental noise.

#### Implementation

Fang et al.^16^ describe the analytic procedure to extract *F*. The intensity ${\tilde{I}}(\lambda ,r,n_{re})$ can be computed using Mie theory with the Python module *miepython*. $\Delta I(\lambda )$ is obtained by subtracting the co-polarized signal (A and P ||, Fig. 10 ) with the cross-polarized signal (A and P $\perp$) after background subtraction and white standard normalization^30^. Experimentally, $\Delta I(\lambda )$ was measured over the range 550–700 nm with 32 spectral points using a hyperspectral camera (HERA VIS-NIR, Nireos). However, the relevant information in the LSS spectra is contained within the low frequencies^16^. We confirmed this observation by injecting the size distributions extracted from the histological analyses into the forward LSS model. Similarly to what was done by Fang et al.^16^, 16 points were kept to resolve the differential polarization signals across the 550–700 nm range. The resulting spectral resolution was 9.3 nm (Fang et al.^16^ used 8.9 nm). Multiple acquisitions were realized to measure the LSS spectra from different positions for each tumor (Table 1). The relative refractive index $n_{re}$ was set to 1.06 to target the nuclei/cytoplasm refractive index variation^16,17^. The surrounding medium refractive index was assumed to be 1.38^13^. During the acquisitions, the samples were submerged in an index matching solution with the same refractive index as the surrounding medium. To minimize the coherent signal in each spectral image, the angular intensity was integrated within a ring spanning from 1.0° to 1.5° for each wavelength. To mitigate the effect of multiple scattering, LSS spectra with a mean degree of polarization DOP (DOP = ($|I_{\parallel }-I_{\perp }|/|I_{\parallel }+I_{\perp }|$) above 0.35 were selected. This threshold lead us to take into account 11 out of 14 measured spectra for the chondrosarcomas and 3 out of 7 spectra for osteosarcomas (MOS-J type only). This part of the image corresponds to what EBS considers as the incoherent baseline. Eq. (8) was multiplied by $\lambda ^4$ prior to differentiation with respect to $\lambda$ to mitigate the influence of Rayleigh scattering^16^. The resulting equation was solved using a linear least squares algorithm with a non-negativity constraint applied to *F*. The minimum radius for the size estimation $r_{min}$ was set to 100 nm. While rationales can be found in the literature to justify the $r_{min}$ value and the number of points to reconstruct *F*, the choices of $r_{max}$ values appear to be predominantly arbitrary. Yet, this parameter has an important physical meaning since it corresponds to the largest scatterer contribution “allowed” in the differential polarization spectrum. Hence, multiple matrices ${\tilde{I}}(\lambda ,r,n_{re})$ with different maximum radii $r_{max}$ were computed. The different $r_{max}$ values were limited to around 17 $\upmu$m to include the chondrosarcoma cells and to ensure a sufficiently fine radius resolution capable of distinguishing the size distribution of osteosarcoma nuclei from that of chondrosarcoma (about one micron).

This study focused on a single $r_{max}$ values because the estimation of the size distribution *F* is extracted from the precomputed LSS spectrum matrix ${\tilde{I}}(\lambda ,r,n_{re})$. The implementation of this look-up table approach results in computational times of only a few seconds, thus enabling real-time inversions. Hence, a unique and common ${\tilde{I}}$ matrix was used to characterize the two tumor types, leading us to the assumption that the relative refractive index between the nuclei and the cytoplasm is equal to the one between cells and the extracellular matrix.

LSS has been initially developed to probe the nucleus size distribution. However, we make the hypothesis that cells could be involved in the scattering process, particularly in media with abundant extracellular matrix. Indeed, Mie Theory describes the interaction of light of discrete spherical scatterers in a homogeneous surrounding medium. Therefore, cells could be considered as scatterers within the extracellular matrix, analogous to how nuclei scatter the electromagnetic incident plane wave within the cytoplasm. Therefore, we took the LSS analysis one step further using the estimated size distributions. To account for both the nucleus and the cell scattering, linear combinations of the nucleus and the cell histological histograms were computed for different nucleus weight values $w_{nuc}$, defined such as $w_{nuc} = 1-w_{cell}$. The optimized nucleus weight was then extracted by minimizing the root mean squared error (RMSE) between the estimated LSS solution *F* and the newly computed histogram for each tumor type.

Cell mitochondria can also scatter light^31^. However, Ghosh et al.^32^ measured mitochondria sizes in sarcoma cells and reported a longest dimension of 161 nm. Given the 700 nm excitation, the *ka* product can be estimated at $ka = 0.7$ at maximum, indicating scattering at the frontier between the Mie and the Rayleigh scattering regions. The Mie scattering of large mitochondria could appear in the estimated scatterer size distribution (minimum radius of 100 nm) while smaller mitochondria are considered as Rayleigh scatterers and therefore have their influence mitigated through post-processing treatment. Consequently, the scattering of mitochondria is not expected to interfere with the nucleus scattering of interest due to their size smaller in comparison.

**Table 1:** Degree of averaging and number of underlying independent measurements per technique. Independent measurement refers to ROI for ultrasound techniques* and sample position for optical techniques^†^. Checkmarks refer to the following number of ROIs: 36 (Ch1), 75 (Ch2), 88 (Ch3), 51 (Ch4), 24 (Ch5), 23 (Os1), 27 (Os2), 61 (Os3), 45 (Os4).

Method	Independent Measurements	Mean per animal	Mean per tumor group
BSC estimations*, Fig. 2		$\checkmark$	
Linear model* (18–38 MHz), Fig. 3 a	$\checkmark$		
ES*, (18–38 MHz), Fig. 3b	$\checkmark$		
${\textrm{EBS}}^{\dagger }$, Fig. 4a		5 per Ch., 1 per Os.	
${\textrm{EBS\, differences}}^{\dagger }$, Fig. 4b			Ch : 5 ; Os(K7M2) : 2 ; Os(MOS-J) : 2
PII* (13–24 MHz), Fig. 5		$\checkmark$	
FFSM* (24–38 MHz), Fig. 6			Ch : 274 ; Os(K7M2) : 50 ; Os(MOS-J) : 106
PII* (13–38 MHz), Fig. 7		$\checkmark$ (Os. only)	
${\textrm{LSS}}^{\dagger}$, Figs. 8 and 9			Ch : 11 ; Os(MOS-J) : 3

## Data Availability

The datasets collected and analyzed during the current study are available from the corresponding author on reasonable request.
