# Author Correction: Cancer characterization using light backscattering spectroscopy and quantitative ultrasound: an ex vivo study on sarcoma subtypes

**DOI:** 10.1038/s41598-023-46961-9

**Published:** 2023-11-14

**Authors:** Cyril Malinet, Bruno Montcel, Aurélie Dutour, Iveta Fajnorova, Hervé Liebgott, Pauline Muleki-Seya

**Affiliations:** 1 Université de Lyon, CREATIS, CNRS UMR 5220, Inserm U1044, INSA-Lyon, Université Lyon 1, Lyon, France; 2 Centre de Recherche en Cancérologie de Lyon/Centre Léon Bérard, Equipe mort cellulaire et cancers pédiatriques, UMR INSERM 1052, CNRS 5286, Lyon , France

Correction to: *Scientific Reports* 10.1038/s41598-023-43322-4, published online 03 October 2023

The original version of this Article contained errors in the legends of Figures 8 and 9.

Figure 8: (**a**) Mean differential polarization signals ± standard error for the two tumor types. (**b**) and (**c**) Estimated scatterer size distribution for the chondrosarcoma and the osteosarcoma respectively. The nuclear and cellular size distribution estimated from histological analyses are normalized.

now reads:

Figure 8: (**a**) Mean differential polarization signal ± standard error for the two tumor types. (**b**) and (**c**) Estimated scatterer size distribution for the osteosarcoma and the chondrosarcoma respectively. The nuclear and cellular size distributions estimated from histological analyses are normalized.

Figure 9: (**a**) Chondrosarcoma estimated solution and its optimized linear combination of nucleus and cell size distribution. The cell scattering identified in the estimated solution accounts for 52%. (**b**) Osteosarcoma estimated solution and its optimized linear combination of nucleus and cell size distribution. The nucleus scattering identified in the estimated solution accounts for 69%.

now reads:

Figure 9: (**a**) Osteosarcoma estimated solution and its optimized linear combination of nucleus and cell size distribution. The nucleus scattering identified in the estimated solution accounts for 69%. (**b**) Chondrosarcoma estimated solution and its optimized linear combination of nucleus and cell size distribution. The cell scattering identified in the estimated solution accounts for 52%.

The original article has been corrected.

