# Author Correction: Cancer characterization using light backscattering spectroscopy and quantitative ultrasound: an ex vivo study on sarcoma subtypes

**DOI:** 10.1038/s41598-024-56927-0

**Published:** 2024-03-18

**Authors:** Cyril Malinet, Bruno Montcel, Aurélie Dutour, Iveta Fajnorova, Hervé Liebgott, Pauline Muleki-Seya

**Affiliations:** 1 Université de Lyon, CREATIS, CNRS UMR 5220, Inserm U1044, INSA-Lyon, Université Lyon 1, Lyon, France; 2 Centre de Recherche en Cancérologie de Lyon/Centre Léon Bérard, Equipe mort cellulaire et cancers pédiatriques, UMR INSERM 1052, CNRS 5286, Lyon , France

Correction to: *Scientific Reports* 10.1038/s41598-023-43322-4, published online 03 October 2023

The original version of this Article contained an error in Figure 2, where the Y-axis labels were incorrect.

The original Figure 2 and accompanying legend appear below.

**Figure 2:**
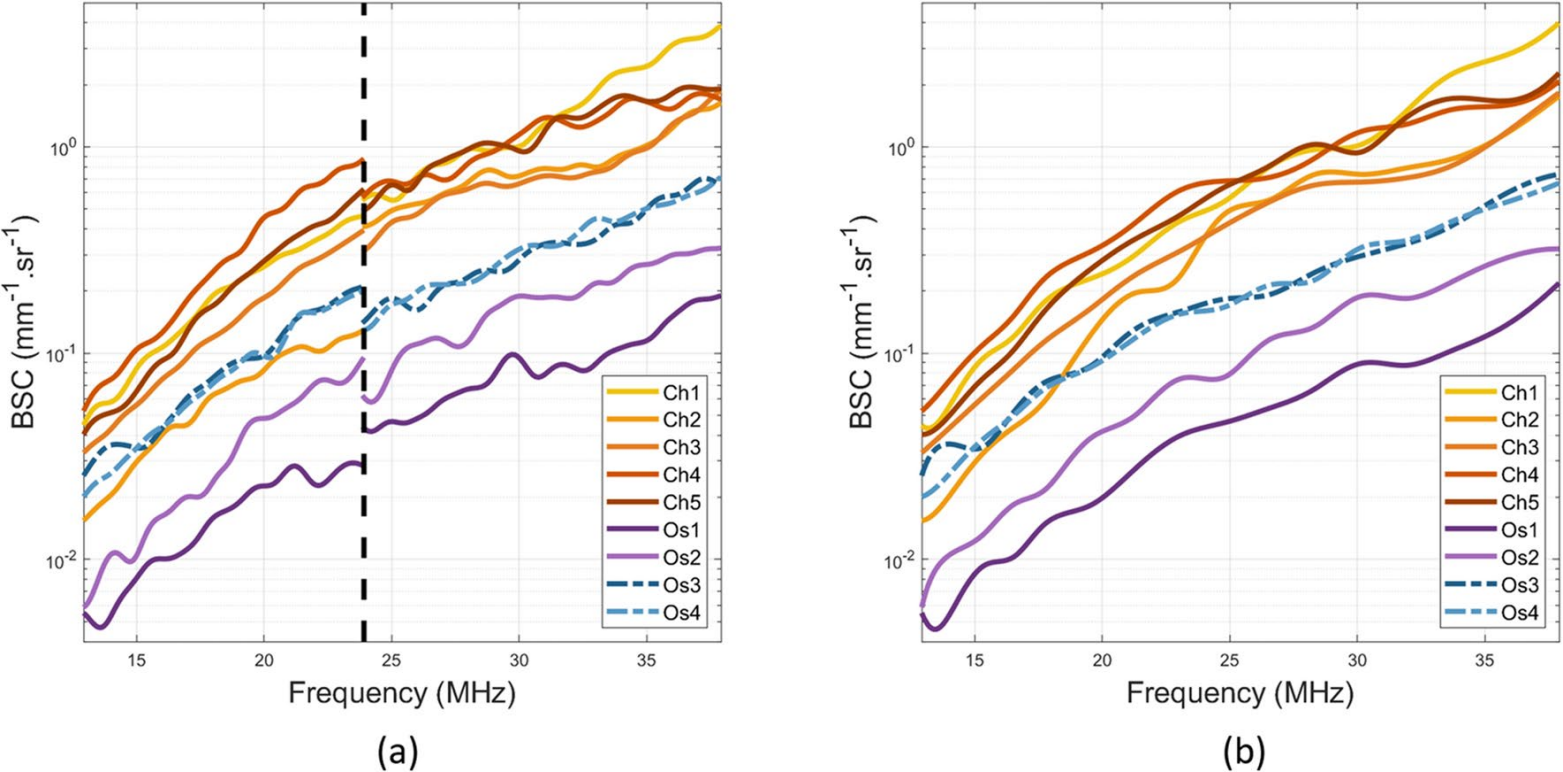
(**a**) Mean estimated backscatter coefficients (BSC) with the MS-250S probe (left of the black dotted line) and the LZ-400 probe (right of the black dotted line) per animal. (**b**) Corresponding BSC b-spline fits. ’Ch’ stands for chondrosarcomas and ’Os’ for osteosarcomas.

The original Article has been corrected.

